# Comparison of the effect of three different protein content enteral diets on serum levels of proteins, nitrogen balance, and energy expenditure in critically ill infants: study protocol for a randomized controlled trial

**DOI:** 10.1186/s13063-019-3686-8

**Published:** 2019-10-11

**Authors:** Reyes Fernández, Javier Urbano, Ángel Carrillo, Ana Vivanco, María José Solana, Corsino Rey, Jesús López-Herce

**Affiliations:** 1 Pediatric Intensive Care Unit, Department of Pediatrics, Hospital Universitario Central de Asturias, Oviedo, Spain; 2 Pediatric Intensive Care Unit, Hospital General Universitario Gregorio Marañón, Gregorio Marañón Health Research Institute (IISGM), Madrid, Spain; 3 Maternal and Child Health and Development Research Network (REDSAMID), Institute of Health Carlos III, Madrid, Spain; 4 Universidad Complutense de Madrid, Madrid, Spain; 5 Universidad de Oviedo, Oviedo, Spain

**Keywords:** Children, Intensive care, Nutrition, Enteral feeding, Protein intake, Protein balance

## Abstract

**Background:**

Nutritional support is essential in the care of critically ill children since malnutrition in this population is associated with increased morbidity and mortality. Injury in patients admitted to pediatric intensive care units (PICU) results in a catabolic state and augmented protein breakdown, leading to a negative protein balance. Current recommendations about protein prescription in the PICU are fundamentally based on expert opinions, and the minimum threshold is 1.5 g/kg per day of protein, although protein needs could be higher in certain subgroups of patients. The main objectives of the present study are to examine whether the administration of a protein-enriched infant formula increases the serum levels of total proteins, albumin, prealbumin, transferrin, and retinol and improves nitrogen balance and to analyze the effect of the high-protein diet on energy expenditure. A secondary objective is to register possible secondary effects of the protein-enriched diet.

**Methods:**

A multicenter prospective randomized controlled trial (RCT) will be performed in three hospitals. Patients meeting inclusion criteria will be randomly allocated to one of three enteral feeding formulae with different protein contents. Blood and urine test, nitrogen balance assessment, and energy expenditure testing by indirect calorimetry will be performed at the beginning of the nutrition regimen and at 24 h, 72 h and 5–7 days after initiation. The sample size for this trial is estimated to be 90 participants (about 30 participants in each group). The data analysis will be by intention to treat.

**Discussion:**

This RCT will provide new data about the amount of protein needed to improve levels of serum protein and nitrogen balance, a surrogate of protein balance, in critically ill infants receiving enteral nutrition.

**Trial registration:**

ClinicalTrials.gov identifier: NCT03901742. Registered April 1, 2019 – Retrospectively registered.

**Electronic supplementary material:**

The online version of this article (10.1186/s13063-019-3686-8) contains supplementary material, which is available to authorized users.

## Background

Nutritional support is an essential aspect in the care for children with critical illness. Malnutrition has been reported with a prevalence of between 24% and 70% of critically ill children, depending on the series [1–5]. It can be present before admission or develop and increase during the hospital stay [6] because of several factors, such as metabolic stress response, inaccurate estimation of energy requirements, and inappropriate nutrient delivery [7]. Protein-caloric malnutrition, which has an incidence of 15–20% [8], is the most important type of malnutrition in pediatric intensive care units (PICUs). It is associated with poor outcomes in critically ill children: malnourished patients present an increased physiologic instability and quantity of care [9] and have a higher duration of mechanic ventilation and length of stay and increased mortality [2, 3, 10].

Injury in the pediatric population admitted to intensive care units results in a catabolic state and increased protein turnover. Hepatic protein synthesis is enhanced, but there is an even more augmented muscle protein breakdown, leading to negative net protein balance (PB), which conduces to a loss of lean body mass [11–13]. The progressive degradation of muscle mass can affect the diaphragm and other muscles involved in respiration, which may contribute to the onset or worsening of respiratory failure and even loss of heart muscle [14]. These changes are observable within the first two weeks of admission [6], but the most pronounced deficit of calories and proteins occurs during the first few days. Infants under the age of two are particularly susceptible because of an intrinsic lack of endogenous stores and greater baseline requirements [12].

Current recommendations about protein prescription in critically ill children are fundamentally based on expert opinions [15] since studies on protein supplementation are scarce and have small sample sizes and heterogeneous patient populations, doses of protein, and route of administration. The American Society of Enteral and Parenteral Nutrition (ASPEN) recommends, in their latest guidelines, a minimum intake of protein of 1.5 g/kg per day for children admitted to the PICU [16], a minimum threshold supported by a systematic review including 347 mechanically ventilated PICU patients [17] and a cohort study with 76 subjects [18].

Higher protein intake has been associated with early achievement of positive nitrogen balance (NB), a surrogate of PB [19–21], promoting protein anabolism [22]. Moreover, higher protein delivery has been related to lower mortality and higher ventilator-free days in PICU patients [23, 24]. However, the exact and safe amount of protein needed to avoid negative PB remains unclear. Protein intakes greater than 3 g/kg per day have been associated with elevated serum urea nitrogen levels and metabolic acidosis [19, 20, 25, 26], but other studies have reported a higher incidence of lower intelligence quotient in very-low-birth-weight infants receiving over 6 g/kg per day of protein [27, 28].

The aim of the present study is to analyze whether the administration of a high-protein diet improves protein metabolism (serum protein levels and NB) in critically ill infants without increasing energy expenditure assessed by indirect calorimetry. A secondary objective is to register possible secondary effects of the protein-enriched diet.

## Methods

### Hypothesis and aims of the study

The hypotheses of this study are that (1) critically ill infants receiving a higher amount of proteins on enteral feeding will experience a higher increase on serum protein levels—total proteins, albumin, prealbumin, transferrin, and retinol binding protein (RBP)—and NB than children receiving a standard enteral diet, (2) the administration of an enriched protein and high-protein-enriched diet does not increase energy expenditure in critically ill infants, and (3) protein-enriched and high-enriched protein enteral diets are well tolerated, and mild gastrointestinal side effects and tolerable serum urea and total protein elevation are adverse events.

The corresponding objectives are (1) to analyze whether the administration of a higher amount of protein on enteral diet improves protein metabolism observed by the increase of specific serum protein levels (total proteins, albumin, prealbumin, transferrin, and RBP) and NB in critically ill infants, (2) to evaluate the effect of protein supplementation on energy expenditure in these patients, and (3) to register possible secondary effects of the administration of protein-enriched and high-enriched protein diet.

### Study design

**Fig. 1:**
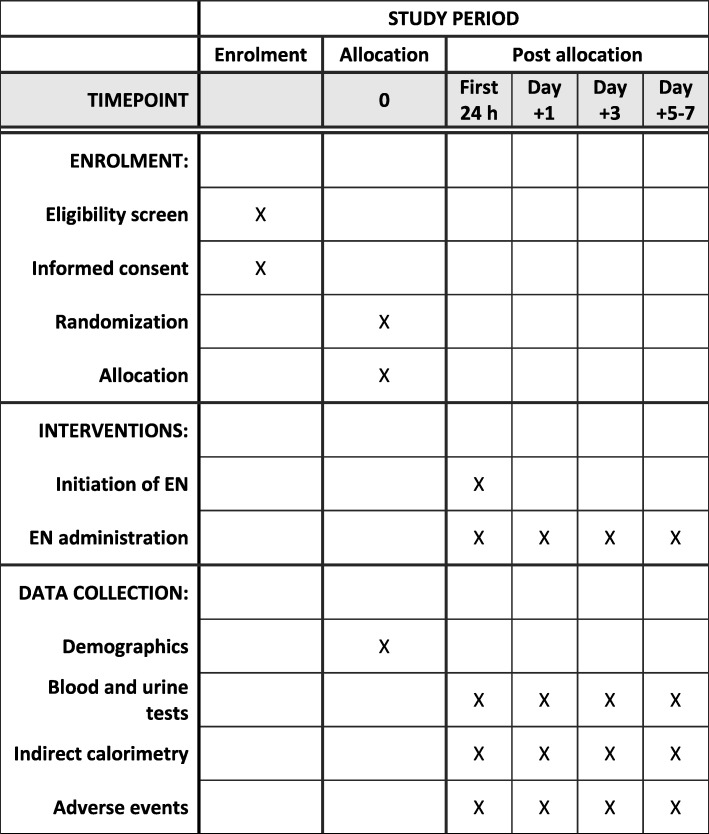
Standard Protocol Items: Recommendations for Interventional Trials (SPIRIT) diagram for this protocol

**Fig. 2:**
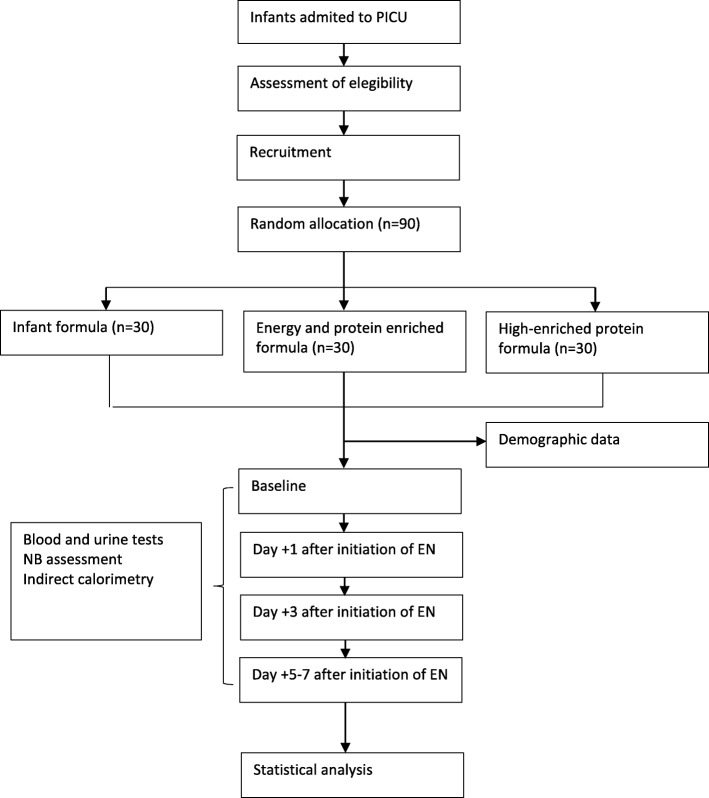
Flowchart for this protocol, comparing the effect over protein metabolism of three different amounts of protein delivery. *Abbreviations*: *EN* enteral nutrition, *NB* nitrogen balance, *PICU* pediatric intensive care unit.

This is an open randomized controlled clinical trial. The study was conceived and designed in 2010. It has been retrospectively registered on the Clinical Trials Database (ClinicalTrials.gov) with the registry number NCT03901742. This protocol, version 1.0, was approved on September 30, 2010. Central ethical approval has been confirmed from the institutional review board of Hospital General Universitario Gregorio Marañón (reference approval number 2010–022851-47), and recruiting will not begin at other centers in the trial until local ethical approval has been obtained. Informed consent will be obtained from parents before their children are enrolled in the study. A report releasing study results will be submitted for publication in an appropriate journal. This protocol has been written in accordance with the Standard Protocol Items: Recommendations for Interventional Trials (SPIRIT) (Fig. 1). The study flowchart is shown in Fig. 2. A SPIRIT checklist is provided in Additional file 1.

### Study setting

PICUs from three hospitals in Spain will participate: Hospital General Universitario Gregorio Marañón (Madrid), Hospital Clínico Universitario de Santiago (Santiago de Compostela), and Hospital Universitario Central de Asturias (Oviedo).

### Participants

#### Inclusion criteria

The following are the inclusion criteria:

1. children 1 month to 2 years old
2. children admitted to the PICU
3. children receiving enteral nutrition with an estimated length of over 72 h.

#### Exclusion criteria

Children who met any of the following criteria will be excluded:

1. age less than 1 month or more than 2 years
2. diabetes mellitus or any inborn metabolic error
3. parenteral nutrition
4. bicarbonate infusion
5. renal replacement therapy
6. children receiving exclusive breastfeeding or in need of special enteral formula.

### Recruitment, randomization, and study development

Once an eligible patient is admitted to the PICU, written informed consent will be requested from parents or legal representative of the child by the physician responsible for the patient. They will be made aware that participation is voluntary, and they will be allowed to refuse further participation in the trial whenever they want.

After enrollment, the patient will be allocated randomly, in order of recruitment, into one of the three diet groups by using a randomized data table generated with EPIDAT 3.1 software (Servicio de Información sobre Saúde Pública de la Dirección Xeral de Saúde Pública de la Consellería de Sanidade, Xunta de Galicia, in collaboration with the Health Information and Analysis Unit of the Pan American Health Organization (PAHO-WHO)). A copy of the randomization list will be securely stored in an envelope located in a PICU working area desk drawer, which will be opened after patient enrollment in the study. Physicians, care givers, and investigators will know the allocation prior to the start of enteral feeding.

All patients will receive exclusively enteral nutrition via a nasogastric or transpyloric tube. The Standard Enteral Nutrition (SEN) group will be fed exclusively with cow’s milk–based infant formula (Nidina 1; Nestlé, Barcelona, Spain). The Protein-enriched Enteral Nutrition (PEN) group will be fed exclusively with a polymeric infant formula (Infatrini; Nutricia, Madrid, Spain). The High Protein-enriched Enteral Nutrition (HPEN) group will receive a polymeric infant formula (Infatrini; Nutricia) supplemented with 2.6 g of protein/100 mL of formula. The source of the protein supplement will be a non-hydrolyzed protein cow’s milk–based formula (Resource Protein Instant; Nestlé) (Table 1).

**Table 1:** Composition of diets used in the study

	Protein (g)	Carbohydrate (g)	Lipids (g)	Energy (kcal)
Standard enteral nutrition	1.7	7.4	3.4	67
Protein-enriched enteral nutrition	2.6	10.3	5.4	100
High protein-enriched enteral nutrition	5.1	10.5	5.5	110

Content per 100 mL

Since this is an open-label trial, the assigned diet will be written down on the medical prescription of each patient and will be prepared by the PICU staff at their own unit, using for it the branded bottles where the different formulae are commercialized.

Continuous enteral nutrition will be typically initiated within the first 24 h of PICU admission, by a transpyloric or nasogastric tube, at a rate of 0.5–1 mL/kg per hour, with increases of 0.5–1 mL/kg every 3–4 h, if well tolerated, to reach a caloric intake of 60–65 kcal/kg per day or as needed on the basis of resting energy expenditure measured by indirect calorimetry. There is no limit of time before the patient must be recruited as long as it is before the enteral feeding is initiated. The establishment of enteral nutrition should never be delayed by achieving enrollment.

Demographic data—gender, age, weight, height, and diagnosis on admission—will be recorded at inclusion. The risk of mortality at admission will be calculated by using pediatric scales: Pediatric Index of Mortality 2 (PIM2), Pediatric Risk of Mortality (PRISM), and Pediatric Logistic Organ Dysfunction (PELOD).

Blood concentrations of urea, creatinine, total proteins, albumin, prealbumin, transferrin, RBP levels, urinary concentration of urea in 24 h or isolated urine sample, and energy expenditure, oxygen consumption (VO_2_), and carbon dioxide production (VCO_2_) by indirect calorimetry (Datex S5 monitor, E-COVX; GE Healthcare/Datex-Ohmeda, Helsinki, Finland) will be measured at admission and at days 1, 3, and 5–7 after initiation of enteral feeding. Air leaks will be measured by using the mechanical ventilator. Calorimetry-derived data will be collected only in patients who meet all the following requirements: tracheal intubation, air leakage less than 10 %, fraction of inspired oxygen (FiO2) less than 80%, not treated with inhaled medicinal gases (nitric oxide, sevofluorane or heliox), and not connected to extracorporeal membrane oxygenation (ECMO). The collection of indirect calorimetry data will be carried out over 30 to 120 min.

NB will be calculated as nitrogen intake minus total nitrogen losses. Total nitrogen losses will include total urinary nitrogen and fecal/miscellaneous losses estimated in accordance with the World Health Organization recommendations [29]. Other blood biochemical parameters such as glucose, cholesterol, triglycerides, procalcitonin, C-reactive protein, ions, and blood gas will also be recorded.

Time between PICU admission and the initiation of enteral nutrition, total daily enteral energy and protein delivery, intravenous albumin infused, and other treatments such as vasoactive drugs, neuromuscular blockers, sedatives and analgesic drugs, diuretics, and steroids will be registered.

### Protein-enriched diet safety

Enteral complications (constipation, diarrhea, abdominal distension, and gastric residue), serum urea and total protein levels as well as any unexpected adverse event occurring during the trial will be recorded to evaluate the safety of the protein-enriched diet. The discontinuation of the enteral nutrition will be decided on the judgment of the physician looking after these patients.

### Study ending

The study will be ended:

1. after 7 days of enteral feeding.
2. at PICU discharge.
3. if hyperproteinemia greater than 8.5 g/dL is present.
4. if serum urea levels exceed 80 mg/dL without evidence of renal function disturbance—in accordance with KDIGO (Kidney Disease Improving Global Outcomes) criteria—or suspected hypercatabolic state (underlying condition and negative nitrogen balance).

### Study outcomes

The primary outcome of the study will be the variation of NB from baseline to the study ending and the incidence of hyperproteinemia or uremia causing the need to stop the study. Secondary outcomes include the variation of plasma protein levels (total proteins, albumin, prealbumin, transferrin, and RBP), expenditure energy measured by indirect calorimetry, and the incidence of gastrointestinal complications (abdominal distension, vomiting, diarrhea, and excessive gastric residue) and metabolic acidosis.

### Data management

Trial data will be extracted from the medical history of the patient, registered by investigators on a data collection form, and recorded on a central database. Each patient will be identified by number of medical history and subject number, so confidentiality of the patient will be kept.

### Statistical analysis

As there are no previous studies reporting expected standard deviations, we use the standardized difference of means for computing the optimal minimum number of patients to include in the trial. The calculation of the sample size was carried out with EPIDAT 3.1 software. Given a significance level of 5% (type I error), a power of 80% (complementary of the type II error), and a minimum detectable standardized difference of means of 0.9, we need 30 patients per group (Bonferroni correction included).

We will use an intention-to-treat approach. A descriptive analysis will be performed where quantitative variables will be described by their means and standard deviations or their medians and interquartile ranges, as appropriate. The quantitative ones will be summarized by their absolute and relative frequencies. Parametric and non-parametric tests will be employed for contrasting equality among groups.

Univariate and multivariate mixed models will be used in order to assess the effects size of the different diets (fixed effects) on the patients (random effects) unadjusted and adjusted by potential confounders, respectively. *P* values under 5% will be considered statistically significant.

## Discussion

Current recommendations about protein prescription in critically ill children are not based on randomized controlled trial (RCT) results. Data regarding protein supplementation are lacking. Studies with small sample sizes and heterogeneous patient populations are the only source for this information. A minimum intake of protein of 1.5 g/kg per day for children admitted to the PICU [16] is the current recommendation of the ASPEN in their latest guidelines. This recommendation is supported by only a systematic review [17] and a cohort study [18].

The exact and safe amount of protein needed to avoid negative PB remains unclear. Higher protein intake has been associated with early achievement of positive NB [19–21], and higher protein delivery has been related to lower mortality and higher ventilator-free days in PICU patients [23, 24]. On the other hand, enriched protein diets have been associated with elevated serum urea nitrogen levels [19, 20, 25, 26].

This RCT is designed to obtain new data about the amount of protein needed to avoid negative PB and the amount of protein that is safe without producing secondary effects. The results of the study will be important to decide whether a protein-enriched diet will or will not be useful in critically ill infants. Moreover, the results will be clinically significant to know the exact amount of protein we can recommend for critically ill infants.

In clinical practice, there is no method that can directly evaluate the protein metabolism. On the other hand, NB and serum protein levels have been used in most previous studies to estimate an adequate protein intake. We will use NB, considering a positive NB as an indicator of protein anabolism and a negative NB as protein catabolism. Moreover, we will evaluate as an indirect indicator of a protein metabolism improvement the increase of total proteins, albumin, prealbumin, RBP, and transferrin serum levels from baseline to the study endpoint.

The main problems we will face during data collection are the possible development, in any of the groups, of side effects that recommend to stop the study and the difficulty of including enough valid patients due to problems with urine collection.

In summary, this multicenter, prospective RCT will compare the effect of three different enriched-protein diets on serum levels of proteins, nitrogen balance, and energy expenditure in critically ill infants as well as their possible secondary effects.

## Trial status

The trial began recruitment at Hospital Gregorio Marañón on December 28, 2016. Participants will be recruited until December 2020 if necessary. During the period between approval of the protocol version (2010) and enrollment of the first patient (2016), no protocol amendments or enrollment attempts have occurred.

## Additional file

Additional file 1: The SPIRIT checklist. (DOC 125 kb)

## Data Availability

The final datasets generated and analyzed in the current study will be available from the corresponding author on reasonable request and in ClinicalTrials.gov (NCT03901742).

## Abbreviations

ASPEN: American Society of Enteral and Parenteral Nutrition
NB: Nitrogen balance
PB: Protein balance
PICU: Pediatric intensive care unit
RBP: Retinol binding protein
RCT: Randomized controlled trial
SPIRIT: Standard Protocol Items: Recommendations for Interventional Trials
